# Severe, but not mild to moderate, non-alcoholic fatty liver disease associated with increased risk of subclinical coronary atherosclerosis

**DOI:** 10.1186/s12872-021-02060-z

**Published:** 2021-05-19

**Authors:** Chia-Chi Hsiao, Pai-Hsueh Teng, Yun-Ju Wu, Yi-Wen Shen, Guang-Yuan Mar, Fu-Zong Wu

**Affiliations:** 1 Section of Thoracic and Circulation Imaging, Department of Radiology, Kaohsiung Veterans General Hospital, No. 386, Ta-Chung 1st Road, Kaohsiung, 81362 Taiwan; 2 Department of Medical Imaging and Radiology, Shu-Zen Junior College of Medicine and Management, Kaohsiung, Taiwan; 3 Physical Examination Center, Kaohsiung Veterans General Hospital, Kaohsiung, Taiwan; 4 Faculty of Medicine, School of Medicine, National Yang-Ming University, Taipei, Taiwan; 5 Department of Medical Education and Research, Kaohsiung Veterans General Hospital, Kaohsiung, Taiwan

**Keywords:** Fatty liver, Coronary artery atherosclerosis, Vulnerable plaque(s)

## Abstract

**Background:**

Non-alcoholic fatty liver disease (NAFLD) is associated with high risk of cardiovascular disease. The prevalence is increasing to 45–65% in the general population with routine health check-up, and most subjects have the mild degree NAFLD in recent years. Moreover, there are no studies on the association between NAFLD severity and coronary atherosclerosis in the real-world setting by ultrasonography.

**Methods:**

The aim of this study was to determine the relationship between the severity of NAFLD and subclinical coronary atherosclerosis. Overall, 817 subjects meet criteria for NAFLD were enrolled in the retrospective cohort study (155 subjects were excluded). The severity of NAFLD was divided into the normal, mild, moderate and severe degree based on the finding of abdominal ultrasonography. The assessment of coronary atherosclerosis was based on CAC scan/coronary CT angiography finding in terms of CAC score ≧ 100, CAC score ≧ 400, CAD-RADS ≧ 3 and presence of vulnerable plaque(s).

**Results:**

A significant linear trend was observed between the severity of NAFLD and subclinical coronary atherosclerosis. Compared with the reference group (including normal, mild, and moderate NAFLD), severe degree NAFLD was the independently associated risk of subclinical coronary atherosclerosis in term of CAC score ≧ 100, CAC score ≧ 400, CAD-RADS ≧ 3 and presence of vulnerable plaque(s) based on binary logistic regression after adjustment for FRS score and body fat percentage.

**Conclusions:**

Severe degree, but not mild to moderate, was associated with high risk of subclinical coronary atherosclerosis, independently of FRS score and body-fat percentage.

**Supplementary Information:**

The online version contains supplementary material available at 10.1186/s12872-021-02060-z.

## Introduction

Non-alcoholic fatty liver disease (NAFLD) is generally considered as a very common disease in the worldwide at this time. The prevalence of NAFLD in the general population is about 25% in the world, including Western and Asian countries according to recent literature reviews [1, 2]. NAFLD comprises a wide range of different conditions from non-alcoholic fatty liver, non-alcoholic steatohepatitis (NASH) to cirrhosis, and the prevalence is increasing worldwide over time due to eating habits and environment change[3, 4]. NAFLD is highly associated with metabolic syndrome and hepatic insulin resistance, which are considered to play an important role in developing coronary atherosclerosis [5–7]. Recent systemic review/meta-analyses have investigated the association between presence of NAFLD with coronary arteries disease in terms of obstructive coronary artery disease and coronary artery calcification [8–10]. However, there was limited data regarding the relationship between the severity of NAFLD and subclinical coronary artery disease [11, 12]. Therefore, the aim of this study was to determine the relationship between subclinical coronary artery disease and the severity of NAFLD in an Asian population in the real-world setting.

## Methods

### Clinical, anthropometric and laboratory measurements of study cohort

**Fig. 1:**
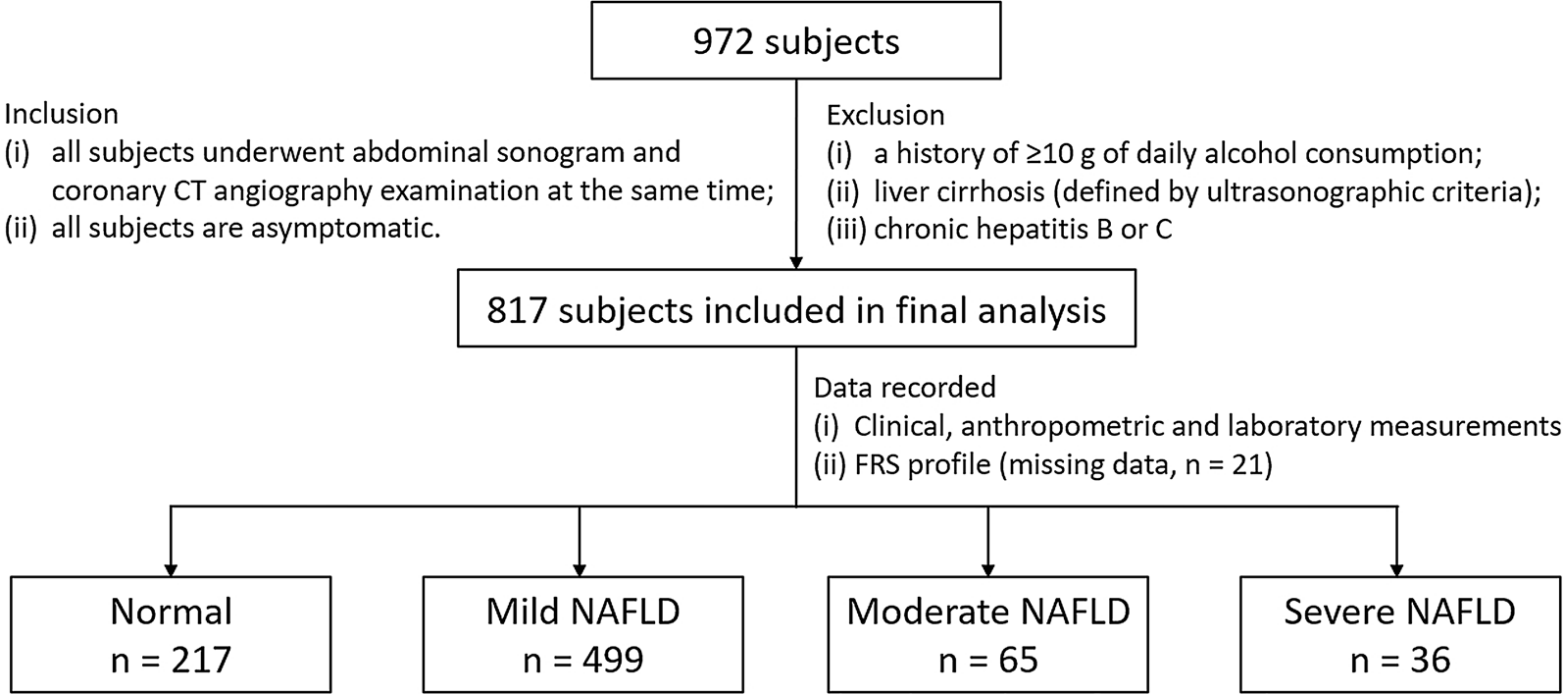
The flowchart of enrollment and inclusion/exclusion criteria for the study subjects

The study protocol was approved by the Institutional Review Board Committee of Kaohsiung Veterans General Hospital, Kaohsiung, Taiwan as No. VGHKS 19-CT6-02. The requirement for informed consent was waived due to retrospective study design by the ethics committee of Kaohsiung Veterans General Hospital. All methods were performed in accordance with the relevant guidelines and regulations. In this retrospective study, we reviewed the records of 972 subjects who underwent routine health checkups, abdominal sonogram and coronary CT angiography examination at the same time in Kaohsiung Veterans General Hospital from January 1, 2018 to December 31, 2018. The inclusion criteria were as follows: (1) all subjects underwent abdominal sonogram and coronary CT angiography examination at the same time; (2) all subjects are asymptomatic. 155 subjects with at least one of the following: (1) a history of ≥ 10 g of daily alcohol consumption; (2) liver cirrhosis (defined by ultrasonographic criteria); (3) chronic hepatitis B or C (defined by history, serum hepatitis B surface antigen, and anti-hepatitis C antibodies) were excluded to meet the diagnostic criteria of NAFLD according to the National Health and Nutrition Examination Survey III criteria [13]. Of the remaining 817 subjects were enrolled into the study population for further analysis show in Fig. 1. Obtained subjects’ demographics, cardiovascular risk factors and anthropometric measurements were recorded from referral visit information in the electronic medical records (EMR) system, including age, gender, body mass index (BMI), hypertension, systolic blood pressure, hypertension medication, diabetes mellitus, current smoking habits, the amount of pack-year, waist circumstance, body-fat percentage, and alcohol intake (number, frequency, and alcohol percentage of drinks per week according to the National Institute on Alcohol Abuse and Alcoholism) were recorded for all participants. Anthropometric measurements such as BMI and body-fat percentage were measured using the electric impedance method analyzer (XSCAN PLUS II; Jawon Medical, Gyeongsan-si, South Korea) with the patients minimally clothed and wearing no socks as the previous study described [14]. Complete biochemical and blood examinations including blood levels of low density lipoprotein-cholesterol (LDL-C), high density lipoprotein-cholesterol (HDL-C), triglyceride (TG), total cholesterol, estimated glomerular filtration rate (eGFR), hemoglobin A1c (HbA1c) concentration were also recorded at the same time. Framingham risk score (FRS) percentage was calculated based on the six coronary risk factors including age, gender, total cholesterol, HDL-C, systolic blood pressure, and smoking habits for conventional cardiovascular risk stratification[15].

### Abdominal ultrasound for NAFLD severity assessment

The diagnosis of severity of NAFLD was based on the ultrasonographic findings established according to the practice guideline of the American Gastroenterology Association [16]. All ultrasonographic studies were performed by two imaging units (GE Logic E9, GE Healthcare, Wauwatosa, WI, USA; Acuson S2000, Siemens, Germany) with a 3.5- to 5-MHz convex probe. The severity of NAFLD was divided into the normal, mild, moderate, and severe degree based on hepatic ultrasonography. Mild fatty liver was defined as slightly increased liver parenchyma echogenicity compared to kidney/spleen with normal visualization of portal vein wall and diaphragm. Moderate fatty liver was defined as the moderate increase of liver parenchyma echogenicity compared to kidney/spleen with mild dimness of portal wall and diaphragm. Severe fatty liver was defined as the high increase of liver parenchyma echogenicity compared to kidney/spleen with poor visualization of portal wall and diaphragm due to elevation of echogenicity [17]. All images were retrospectively reviewed by one single expert radiologist with 10 year experience in ultrasonography. The expert was blinded to the clinical and laboratory data of subjects and unaware of the previously study reported.

### Coronary artery calcium (CAC) scan and coronary CT angiography

All enrolled study subjects underwent initial CAC scans and sequential coronary CT angiography examinations at the same time with 256-slice CT scanner (Revolution CT, GE Healthcare, Milwaukee, USA). We administered oral beta-blockers (metoprolol) if the heart rate exceeded 65 beats per minute an hour before the exams began to start. All subjects received 0.8 mg of sublingual nitroglycerin shortly prior to the CAC scan. The images were analyzed using a dedicated workstation (AW Server, version 3.2, GE Healthcare). All coronary CT angiography examinations were read by two experienced cardiovascular radiologists. Imaging findings of coronary artery stenosis including luminal stenosis severity, plaque composition, and vulnerable plaque(s) characteristics were analyzed according to Coronary Artery Diseases Reporting and Data System (CAD-RADS) classification by 2016 SCCT guideline, including twenty-five coronary segments on the basis of the percentage of cross-sectional area stenosis [18] shown in Additional file 1: Table S1. For CAC score measurements, noncontrast ECG-gated cardiac CT scans were performed based on the Agatston method by using a commercially available CT workstation (AW Server, version 3.2, GE Healthcare). CAC scores were categorized as 4 categories according to the degree of calcification (0–10: minimal; 10–100: mild; 100–400: moderate; > 400: severe) [19–21]. In this study cohort, primary endpoints including CAC score ≧ 100, CAC score ≧ 400, CAD-RADS ≧ 3 and presence of vulnerable plaque(s) were assessed for severity of NAFLD associated with subclinical coronary atherosclerosis. The definition of CAD-RADS ≥ 3 was that presence of significant luminal stenosis 50–69%% in any one of the triple coronary trees. There are four main vulnerable plaque features on coronary CT angiography: (1) positive remodeling (2) low attenuation plaque (3) napkin-ring sign (4) spotty calcium. Presence of at least two high-risk plaque feature (s) can be indicated with the definition of vulnerable plaque (s) (Additional file 1: Table S1).

Subclinical coronary atherosclerosis was defined as the presence of atherosclerotic plaque or coronary artery calcification score (CACS) ≥ 1 in asymptotic subjects.

### Statistical method and analysis

The patient clinical characteristics, anthropometric and laboratory measurements are expressed as mean ± standard deviation (SD) or median (interquartile range, IQR) and frequency (%) for group comparison. Multiple group comparisons were done by analysis of variance (ANOVA) for normally distributed data and by Kruskal–Wallis test for skewed data. We used the post-hoc Bonferroni test to analyze the differences among these four groups. To assess linear trends in the percentage of subclinical atherosclerosis for increasing severity of NAFLD, the general linear model was used to test the linear trend. In the binary logistic regression, the group with normal, mild, and moderate NAFLD was chosen as reference to investigate the relationship between NAFLD severity and coronary atherosclerosis in term of CAC ≧ 100, CAC ≧ 400, CAD-RADS ≧ 3, and presence of vulnerable plaque(s) after adjustment for FRS score and body fat percentage. A *p* value < 0.05 was considered statistically significant.

## Results

### Baseline clinical characteristics of the study participants

We included 817 subjects (mean age 54.20 ± 10.02 years, 60.20% male) with detailed baseline characteristics and anthropometric and laboratory measurements demonstrated. Among 817 study subjects, 600 (73.43%) subjects have different degrees of NAFLD. The baseline characteristics, anthropometric and laboratory measurements of the study cohort subjects (N = 817) classified into 4 groups according to the NAFLD severity are shown in Table 1. There were statistically significant differences across the four groups for the in the clinical characteristics, anthropometric and laboratory measurements and coronary artery finding demonstrated by one-way ANOVA, except for current smoking habits, the amount of pack-year, body-fat percentage, eGFR and LDL-C level. In terms of Bonferroni post-hoc tests (inter-group comparison) are also shown in Table 1.

**Table 1:** Clinical characteristics of study subjects according to the severity of NAFLD

	Total population (n = 817)	Normal (n = 217) Group 0	Mild NAFLD (n = 499) Group 1	Moderate NAFLD (n = 65) Group 2	Severe NAFLD (n = 36) Group 3	*p* value	0 versus 1	0 versus 2	0 versus 3	1 versus 2	1 versus 3	2 versus 3
Age	54.207 ± 10.025	55.687 ± 10.368	53.104 ± 9.831	57.015 ± 8.776	55.500 ± 10.790	0.001	0.009	0.999	0.999	0.018	0.978	0.999
Gender (male)	491 (60.2%)	109 (50.2%)	310 (62.2%)	43 (66.2%)	29 (80.6%)	0.001	0.015	0.124	0.003	0.999	0.175	0.923
BMI (kg/m2)	24.943 ± 3.833	23.421 ± 3.294	25.152 ± 3.799	26.468 ± 3.238	28.419 ± 4.399	< 0.0001	< 0.0001	< 0.0001	< 0.0001	0.039	< 0.0001	0.062
Hypertension	290 (36.1%)	60 (28.3%)	179 (36.2%)	31 (50%)	20 (57.1%)	0.001	0.257	0.010	0.006	0.193	0.074	0.999
Diabetes mellitus	132 (16.4%)	18 (8.5%)	83 (16.7%)	17 (27.4%)	14 (41.2%)	< 0.0001	0.036	0.002	< 0.0001	0.178	0.001	0.463
Smoking habits	233 (29.9%)	55 (26.6%)	149 (31.2%)	18 (29.5%)	11 (34.4%)	0.621	0.999	0.999	0.999	0.999	0.999	0.999
Pack-year	8.35 ± 16.714	6.67 ± 14.761	8.48 ± 16.230	11.31 ± 22.162	12.07 ± 23.011	0.147	0.999	0.365	0.622	0.999	0.999	0.999
Total cholesterol (mg/dL)	202.125 ± 38.277	205.070 ± 39.542	202.927 ± 36.990	192.934 ± 41.894	188.686 ± 38.347	0.025	0.999	0.171	0.112	0.322	0.198	0.999
HDL-C (mg/dL)	47.852 ± 13.445	52.721 ± 14.867	46.669 ± 12.814	44.279 ± 9.715	40.914 ± 9.687	< 0.0001	< 0.0001	< 0.0001	< 0.0001	0.999	0.073	0.999
LDL-C (mg/dL)	119.366 ± 31.624	121.670 ± 31.440	118.033 ± 30.795	122.483 ± 36.765	118.714 ± 35.029	0.455	0.958	0.999	0.999	0.999	0.999	0.999
Triglyceride (mg/dL)	144.561 ± 91.717	112.140 ± 60.813	153.131 ± 86.891	157.410 ± 96.694	200.114 ± 196.220	< 0.0001	< 0.0001	0.003	< 0.0001	0.999	0.016	0.146
HbA1c (%)	6.009 ± 0.972	5.794 ± 0.631	6.020 ± 1.046	6.362 ± 1.005	6.571 ± 1.157	< 0.0001	0.025	< 0.0001	< 0.0001	0.061	0.006	0.999
Body-fat percentage	24.14 ± 7.916	23.69 ± 11.644	24.12 ± 6.064	25.50 ± 5.979	24.58 ± 6.125	0.485	0.999	0.758	0.999	0.999	0.999	0.999
eGFR (mL/min/1.73 m^2^)	83.35 ± 28.18	83.99 ± 47.70	82.73 ± 16.44	83.49 ± 14.93	87.78 ± 16.62	0.999	0.999	0.999	0.999	0.999	0.999	0.999
Framingham risk score (%)						0.018	0.477	0.134	0.062	0.998	0.378	0.999
< 6	213 (26.8%)	69 (32.7%)	129 (26.3%)	11 (18.0%)	4 (11.8%)							
≧ 6	583 (73.2%)	142 (67.3%)	361 (73.7%)	50 (82.0%)	30 (88.2%)							
CAC score ≧ 100	103 (12.6%)	24 (11.1%)	46 (9.2%)	20 (30.8%)	13 (37.1%)	< 0.0001	0.999	< 0.0001	< 0.0001	< 0.0001	< 0.0001	0.999
CAC score ≧ 400	40 (4.9%)	12 (5.5%)	15 (3.0%)	5 (7.7%)	8 (22.9%)	< 0.0001	0.875	0.999	< 0.0001	0.573	< 0.0001	0.004
CAD-RADS ≧ 3	78 (9.6%)	19 (8.8%)	41 (8.2%)	10 (15.4%)	8 (22.2%)	0.015	0.999	0.673	0.066	0.383	0.034	0.999
Vulnerable plaque (s)	119 (14.6%)	29 (13.4%)	63 (12.6%)	16 (24.6%)	11 (30.6%)	0.002	0.999	0.141	0.039	0.058	0.019	0.999

*BMI* body mass index, *HDL-C* high density lipoprotein-cholesterol, *LDL-C* low density lipoprotein-cholesterol, *HbA1c* hemoglobin A1c, *eGFR* estimated glomerular filtration rate, *CAC* coronary artery calcium, *NAFLD* non-alcoholic fatty liver disease, *CAD-RADS* the coronary artery disease-reporting and data system

### Correlations between severity of NAFLD and subclinical coronary atherosclerosis

**Fig. 2:**
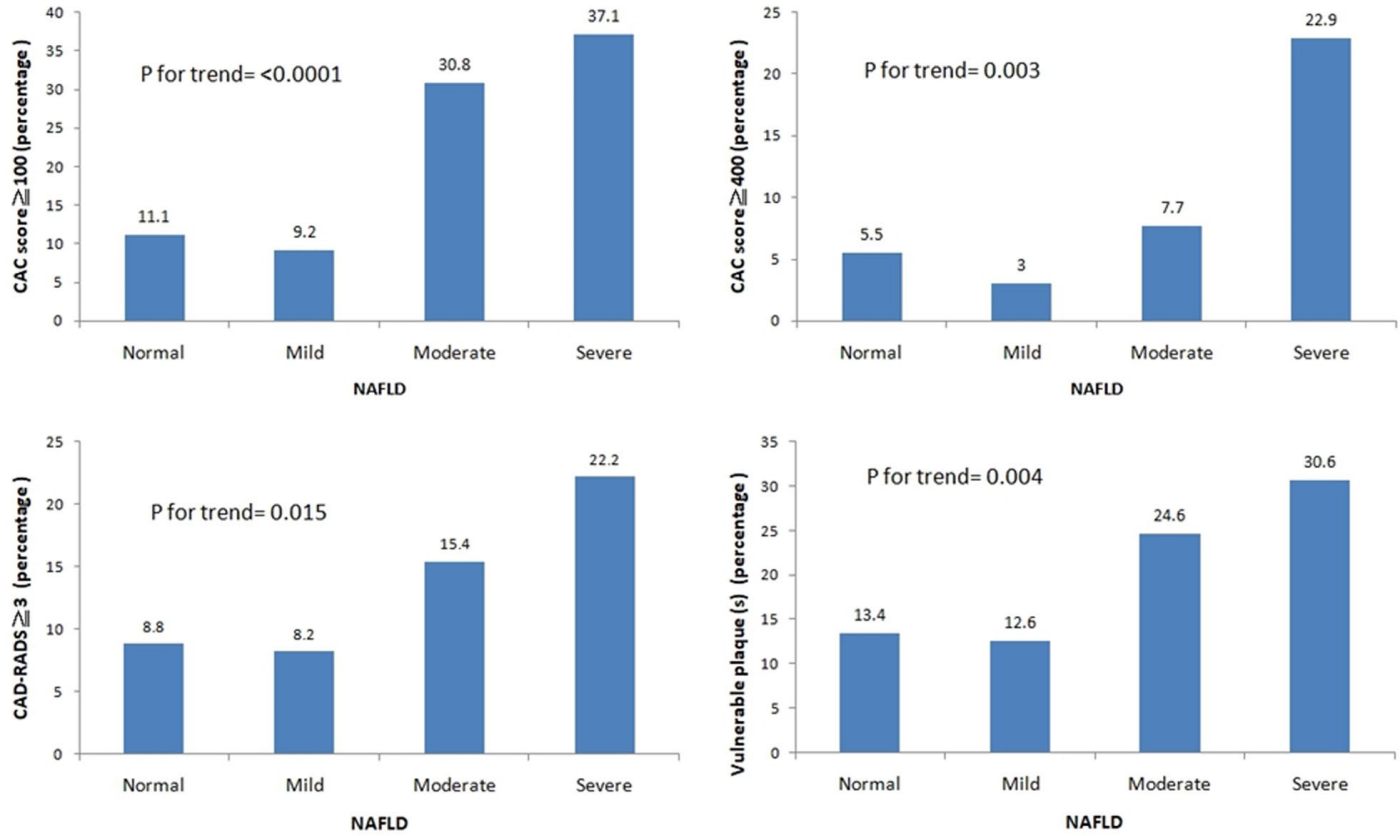
The linear trend of subclinical coronary atherosclerosis according to severity of NAFLD in term of CAC ≧ 100, CAC ≧ 400, CAD-RADS ≧ 3 and presence of vulnerable plaques ( +)

As shown in Fig. 2, subclinical coronary atherosclerosis in term of CAC ≧ 100, CAC ≧ 400, CAD-RADS ≧ 3 and presence of vulnerable plaques ( +) significantly increased as the severity of NAFLD increased by using the general linear model for trend analysis (CAC ≧ 100, *p* for trend < 0.0001; CAC ≧ 400, *p* for trend = 0.003;CAD-RADS ≧ 3, *p* = 0.015; presence of vulnerable plaques, *p* for trend = 0.004.

### Severity of NAFLD associated with subclinical coronary atherosclerosis

Figure 2 shows that the primary endpoints about subclinical coronary atherosclerosis significantly increased as the severity of NAFLD increased by using the general linear model for trend analysis. In addition, post-hoc analysis revealed no significant difference in group 1(mild NAFLD) versus group 2 (moderate NAFLD) and group 0 (normal) versus group 1 (mild NAFLD) in terms of subclinical coronary atherosclerosis. Therefore, in the binary logistic regression, the reference group (including normal, mild NAFLD, moderate NAFLD groups) was chosen as the reference to investigate the relationship between the severity of NAFLD and subclinical coronary atherosclerosis. Table 2 shows the multivariate logistic regression analysis to determine the predictors of subclinical coronary atherosclerosis in term of CAC ≧ 100, CAC ≧ 400, CAD-RADS ≧ 3 and presence of vulnerable plaque(s) with adjustment for FRS score and body-fat percentage. For investigation the association with CAC ≧ 100, severe degree NAFLD is the independent predictor of CAC ≧ 100 after adjusting for FRS score and body-fat percentage (odds ratio of 2.946, 95% CI 1.017–8.530, *p* = 0.046). For investigation the association with CAC ≧ 400, severe degree NAFLD is the independent predictor of CAC ≧ 100 after adjusting for FRS score and body-fat percentage (odds ratio of 4.402, 95% CI 1.149–16.862, *p* = 0.031). For investigation the association with CAD-RADS ≧ 3, severe degree NAFLD is the independent predictor of CAD-RADS ≧ 3 after adjusting for FRS score and body-fat percentage (odds ratio of 4.091, 95% CI 1.396–11.993, *p* = 0.010). For investigation the association with presence of vulnerable plaque(s), severe degree NAFLD is the independent predictor of presence of vulnerable plaque(s) after adjusting for FRS score and body-fat percentage (odds ratio of 2.906, 95% CI 1.107–7.624, *p* = 0.030).

**Table 2:** Multivariable logistic regression models of severe NAFLD on the risk of subclinical atherosclerosis in terms of CAC ≧ 100, CAC ≧ 400, CAD-RADS ≧ 3, and presences of vulnerable plaque(s)

	Model 1 (CAC score ≧ 100)			Model 2 (CAC score ≧ 400)			Model 3 (CAD-RADS ≧ 3)			Model 4 (vulnerable plaque)		
	OR	CI	*p* value	OR	CI	*p* value	OR	CI	*p* value	OR	CI	*p* value
Framingham risk score (%)	1.072	1.038–1.106	< 0.0001	1.096	1.041–1.154	< 0.0001	1.071	1.032–1.110	< 0.0001	1.079	1.050–1.108	< 0.0001
Body-fat percentage	1.005	0.968–1.043	0.789	1.016	0.970–1.064	0.504	1.011	0.976–1.048	0.537	0.981	0.943–1.020	0.330
Severe NAFLD^*^	2.946	1.017–8.530	0.046	4.402	1.149–16.862	0.031	4.091	1.396–11.993	0.010	2.906	1.107–7.624	0.030

*OR* odds ratio, *CI* confidence interval, *NAFLD* Non-alcoholic fatty liver disease, *CAC* coronary artery calcium, *CAD-RADS* the coronary artery disease-reporting and data system

*Reference group normal, mild and moderate NAFLD groups (severe NAFLD vs. reference group)

## Discussion

The focus of the current study is to investigate the relationship between the severity of NAFLD and subclinical coronary atherosclerosis. In this study, we demonstrated three major findings. The first one is that there was no significant difference in terms of subclinical coronary atherosclerosis between the two groups (normal group versus NAFLD group shown in Additional file 1: Table S2). However, a significant difference in term of subclinical coronary atherosclerosis between the two groups observed (the reference group versus severe NAFLD group shown in Additional file 1: Table S3). Second, a significant linear trend was observed between the severity of NAFLD and subclinical coronary atherosclerosis. Third, for investigation of subclinical coronary atherosclerosis in terms of CAC ≧ 100, CAC ≧ 400, CAD-RADS ≧ 3, and presence of vulnerable plaque(s), severe degree NAFLD is the independent predictor of subclinical coronary atherosclerosis after adjusting for FRS score and body fat percentage.

As for the relationship between NAFLD and cardiovascular disease, previous studies including systemic review-metaanalysis have demonstrated that a significant association between NAFLD and subclinical coronary atherosclerosis, obstructive CAD and major adverse cardiovascular events [8–10, 22]. However previous limited studies have investigated the severity degree of NAFLD associated CAD in asymptomatic or symptomatic subjects [11, 12]. In addition, the prevalence of NAFLD has increased from 25–30% in 2012 to 45–65% in 2018 [2, 12, 23]. Recently, the prevalence of lean NAFLD or overweight/obese-NAFLD has grown increasingly [14, 24]. It has become a significant public health concern due to current westernized diets, lifestyle habits change and obesity epidemic in Asia [25]. The prevalence of NAFLD found in our study population was high, reaching 73.43%. Of the 600 subjects with NAFLD, 499 (83.16%) had mild NAFLD, 65 (10.83%) had moderate NAFLD, and 36 (6%) had severe NAFLD. Most subjects with NAFLD are clinically silent and asymptomatic, except for a variable degree of fatigue. This is in line with previous research that shows the prevalence has increased recently.

Therefore, it is very important to address the impact of the different severity of NAFLD on coronary atherosclerosis in the epidemic area of the wide spectrum of NAFLD. Our study showed that severity degree of NAFLD was positively correlated with subclinical coronary atherosclerosis, especially in severe degree NAFLD. This study provides a novel finding that relationship between mild to moderate NAFLD and subclinical coronary atherosclerosis was not significant; however, a positive significant association was demonstrated between severe NAFLD and subclinical coronary atherosclerosis, especially for obstructive CAD. Our study finding could partially explain that few studies analyzing the relationship between NAFLD and CAD, and they present controversial results, which may be due to different spectrums of NAFLD severity [26]. As for the mechanism underlying the positive relationship between severe NAFLD and CAD, abnormal insulin resistance, inflammation with oxidative stress and endothelial dysfunction may play an important pathway for the development of atherosclerosis with a potential dose–response relationship [5, 6, 27].

It has recently been observed that the eradication of HCV (hepatitis C virus) infection with direct-acting antiviral therapy, probably through the elimination of chronic inflammation due to the infection, leads to a reduction in insulin-resistance as well as both the onset of diabetes [28, 29]. These results indirectly confirm the potential mechanism of insulin resistance in the relationship between NAFLD and atherosclerotic disease.

The study has several limitations. First, this retrospective cohort study was cross-sectional design, which limits its cause-and-effect conclusion between severity of NAFLD and subclinical coronary atherosclerosis. In the severe NAFLD group, 41.2% of subjects had diabetes. Therefore, this patient setting was mostly affected by insulin resistance with increasing the risk of atherosclerosis.

In addition, we didn’t collect the C-reactive protein (CRP) level, medication history (such as statins, anti-platelet agents, and Glucagon-like peptide-1 agonists), insulin-mediated cytokine and coronary segment severity score. Therefore, the underlying mechanism assessing the association of the level of CRP, the severity of NAFLD, and subclinical coronary atherosclerosis could not be addressed in this study [30–36]. Second, our retrospective cohort subjects were self-referred, suggestive of a potential selection bias. Third, there are some shortcomings such as operator-dependent and subjective evaluation of NAFLD by ultrasonography [37]. However, the meta-analysis has demonstrated that ultrasonography allows for reliable and accurate diagnosis of moderate-severe fatty liver [38]. In addition, ultrasonography is a currently low cost, non-invasive and easily feasible diagnostic tool for screening for NAFLD in real-world settings. Fourth, this study cohort is based on the population in Taiwan. Therefore, it is not be generalized to another different racial and ethnic population due to differences in health status, dietary habits, and lifestyle. In addition, our study primary endpoint is to investigate the association of fatty liver with coronary atherosclerosis, independently of conventional cardiovascular risks (FRS percentile). The impact of gender difference would not be investigated due to relative small sample size in the severe NAFLD group. Fifth, previous studies/guidelines did not recommend cardiac CT angiography as a screening test in asymptomatic low-risk subjects [39, 40]. However, several studies have retrospectively demonstrated that male gender, diabetes mellitus, and smoking amount are predictors of subclinical coronary atherosclerosis or non-calcified plaque, even in young adults or health checkup populations [41–44]. Our study used a similar retrospective cohort design to investigate the association between the degree of fatty liver and coronary atherosclerosis, independently of traditional cardiovascular risk factors (FRS percentile). However, there are still a small number of missing values existed in FRS profile (N = 21). Finally, this study tried to investigate the relationship between fatty liver and coronary atherosclerosis. Future studies are warranted to investigate the prediction model including conventional cardiovascular risk factors and severity of NAFLD for coronary atherosclerosis prediction [45].

## Conclusion

In conclusion, our results indicate that severity of NAFLD is closely associated with subclinical coronary atherosclerosis, as assessed by coronary CTA and CAC scan, regardless of FRS score and body-fat percentage. This real-world work provides evidence that severe degree, but not mild to moderate NAFLD associated high risk of obstructive CAD. Accordingly, we recommend that physicians should actively care for subjects with the severe degree NAFLD and offer information about high risk of coronary atherosclerosis in the real-world ultrasound health-checkup setting. In the future, the prediction model including conventional cardiovascular risk factors and severity of NAFLD warranted further investigation.

## Supplementary Information

**Additional file 1: Supplement Table 1**. Definition of CAD-RADS category system and vulnerable plaque(s). **Supplement Table 2**. Clinical characteristics of study subjects according to the severity of with or without NAFLD. **Supplement Table 3**. Clinical characteristics of study subjects according to the reference group and severe NAFLD group

## Data Availability

The datasets used and/or analyzed during the current study are available from the corresponding author on reasonable request.
