# Supplementary material for: Severe, but not mild to moderate, non-alcoholic fatty liver disease associated with increased risk of subclinical coronary atherosclerosis
Source: BMC Cardiovasc Disord. 2021 May 19;21:244. doi: 10.1186/s12872-021-02060-z (PMC8132380; doi:10.1186/s12872-021-02060-z)
Supplement: Supplementary file 1 — Additional file 1: Supplement Table 1. Definition of CAD-RADS category system and vulnerable plaque(s). Supplement Table 2. Clinical characteristics of study subjects according to the severity of with or without NAFLD. Supplement Table 3. Clinical characteristics of study subjects according to the reference group and severe NAFLD group [file 12872_2021_2060_MOESM1_ESM.docx]

**Title**

Severe, but not mild to moderate, non-alcoholic fatty liver disease associated with increased risk of subclinical coronary atherosclerosis

**Authors:**

Chia-Chi Hsiao^1,2^, Pai-Hsueh Teng^1,2^, Yun-Ju Wu^1^, Yi-Wen Shen^1^, Guang-Yuan Mar^3^, Fu-Zong Wu^1,2,4,*^

**Author Affiliations:**

^1^Department of Radiology, Kaohsiung Veterans General Hospital, Kaohsiung, Taiwan

^2^Department of Medical Imaging and Radiology, Shu-Zen Junior College of Medicine and Management, Kaohsiung, Taiwan

^3^Physical Examination Center, Kaohsiung Veterans General Hospital, Kaohsiung, Taiwan

^4^Faculty of Medicine, School of Medicine, National Yang-Ming University, Taipei, Taiwan

^5^Department of Medical Education and Research, Kaohsiung Veterans General Hospital, Kaohsiung, Taiwan

^*^Corresponding author:

Fu-Zong Wu, MD, Section of Thoracic and Circulation Imaging Department of Radiology, Kaohsiung Veterans General Hospital, Taiwan No.386, Ta-Chung 1st Road, Kaohsiung, Taiwan 81362; Department of Medical Imaging and Radiology, Shu-Zen Junior College of Medicine and Management, Kaohsiung, Taiwan; Faculty of Medicine, School of Medicine, National Yang-Ming University, Taipei, Taiwan; Department of Medical Education and Research, Kaohsiung Veterans General Hospital, Kaohsiung, Taiwan

| Supplement Table 1. Definition of CAD-RADS category system and vulnerable plaque(s) | | |
| --- | --- | --- |
| **CAD-RADS categories** | | |
| Category | Stenosis | Interpretation |
| 0 | 0% | Absence of CAD |
| 1 | 1-24% | Minimal non-obstructive CAD |
| 2 | 25-49% | Mild non-obstructive CAD |
| 3 | 50-69% | Moderate stenosis |
| 4A | 70-99% single or 2- vessel | Severe stenosis |
| 4B | Left main＞50% or 3- vessel ≧70% |  |
| 5 | 100% | Total coronary occlusion |
| **Four main vulnerable plaque features on coronary CT angiography*** | | |
| 1. Positive remodeling | | |
| 2. Low attenuation plaque | | |
| 3. Napkin-ring sign | | |
| 4. Spotty calcium | | |
| * Presence of at least two high-risk plaque feature(s) can be indicated with the definition of vulnerable plaque(s). | | |

| Supplement Table 2 Clinical characteristics of study subjects according to the severity of with or without NAFLD | | | | |
| --- | --- | --- | --- | --- |
|  | Total population (n=817) | NAFLD (-) (n=217) | NAFLD (+) (n=600) | P-value |
| Age | 54.207 ± 10.025 | 55.687 ± 10.368 | 53.672 ± 9.852 | 0.011 |
| Gender (male) | 491 (60.2%) | 109 (50.2%) | 382 (63.8%) | <0.0001 |
| BMI (kg/m2) | 24.943 ± 3.833 | 23.421 ± 3.294 | 25.490 ± 3.868 | <0.0001 |
| Hypertension | 290 (36.1%) | 60 (28.3%) | 230 (38.9%) | 0.006 |
| Diabetes | 132 (16.4%) | 18 (8.5%) | 114 (19.3%) | <0.0001 |
| Current smoking | 233 (29.9%) | 55 (26.6%) | 178 (31.2%) | 0.215 |
| Pack-year | 8.35 ± 16.714 | 6.67 ± 14.761 | 8.96 ± 17.345 | 0.094 |
| Total cholesterol (mg/dL) | 202.125 ± 38.277 | 205.070 ± 39.542 | 201.056 ± 37.784 | 0.188 |
| HDL-C (mg/dL) | 47.852 ± 13.445 | 52.721 ± 14.867 | 46.081 ± 12.438 | <0.0001 |
| LDL-C (mg/dL) | 119.366 ± 31.624 | 121.670 ± 31.440 | 118.526 ± 31.676 | 0.212 |
| Triglyceride (mg/dL) | 144.561 ± 91.717 | 112.140 ± 60.813 | 156.355 ± 98.049 | <0.0001 |
| HbA1c (%) | 6.009 ± 0.972 | 5.794 ± 0.631 | 6.087 ± 1.059 | <0.0001 |
| Body-fat percentage | 24.14 ± 7.916 | 23.69 ± 11.644 | 24.30 ± 6.062 | 0.363 |
| eGFR (mL/min/1.73 m^2^) | 83.35 ± 28.18 | 83.99 ± 47.70 | 83.12 ± 16.32 | 0.698 |
| Framingham risk score (%) |  |  |  | 0.023 |
| ＜ 6 | 213 (26.8%) | 69 (32.7%) | 144 (24.6%) |  |
| ≧ 6 | 583 (73.2%) | 142 (67.3%) | 441 (75.4%) |  |
| CAC score ≧100 | 103 (12.6%) | 24 (11.1%) | 79 (13.2%) | 0.414 |
| CAC score ≧400 | 40 (4.9%) | 12 (5.5%) | 28 (4.7%) | 0.621 |
| CAD-RADS ≧3 | 78 (9.6%) | 19 (8.8%) | 59 (9.8%) | 0.657 |
| Vulnerable plaque (s) | 119 (14.6%) | 29 (13.4%) | 90 (15.0%) | 0.558 |
| Abbreviation: BMI: body mass index; HDL-C: high density lipoprotein-cholesterol; LDL-C: low density lipoprotein-cholesterol; HbA1c: hemoglobin A1c; eGFR: estimated glomerular filtration rate; CAC: coronary artery calcium; NAFLD: Non-alcoholic fatty liver disease; CAD-RADS: The Coronary Artery Disease-Reporting and Data System | | | | |

| Supplement Table 3 Clinical characteristics of study subjects according to the reference group and severe NAFLD group* | | | | |
| --- | --- | --- | --- | --- |
|  | Total population (n=817) | Reference group (n=781) | Severe NAFLD group (n=36) | P-value |
| Age | 54.207 ± 10.025 | 54.15 ± 9.992 | 55.50 ± 10.790 | 0.429 |
| Gender (male) | 491 (60.2%) | 462 (59.1%) | 29 (80.5%) | 0.011 |
| BMI (kg/m2) | 24.943 ± 3.833 | 24.782 ± 3.730 | 28.419 ± 4.398 | <0.001 |
| Hypertension | 290 (36.1%) | 270 (33.6%) | 20 (55.5%) | 0.008 |
| Diabetes | 132 (16.4%) | 118 (15.1%) | 14 (38.8%) | <0.001 |
| Current smoking | 233 (29.9%) | 222 (28.4%) | 11 (30.5%) | 0.577 |
| Pack-year | 8.35 ± 16.714 | 8.20 ± 16.419 | 12.07 ± 23.011 | 0.222 |
| Total cholesterol (mg/dL) | 202.125 ± 38.277 | 202.734 ± 38.186 | 188.686 ± 38.346 | 0.034 |
| HDL-C (mg/dL) | 47.852 ± 13.445 | 48.167 ± 13.511 | 40.914 ± 9.687 | 0.002 |
| LDL-C (mg/dL) | 119.366 ± 31.624 | 119.395 ± 31.485 | 118.714 ± 35.029 | 0.901 |
| Triglyceride (mg/dL) | 144.561 ± 91.717 | 142.039 ± 83.352 | 200.114 ± 196.220 | <0.001 |
| HbA1c (%) | 6.009 ± 0.972 | 5.983 ± 0.955 | 6.571 ± 1.156 | <0.001 |
| Body-fat percentage | 24.14 ± 7.916 | 24.12 ± 7.996 | 24.58 ± 6.125 | 0.746 |
| eGFR (mL/min/1.73 m^2^) | 83.35 ± 28.18 | 83.149 ± 28.595 | 87.786 ± 16.622 | 0.335 |
| Framingham risk score (%) |  |  |  | 0.044 |
| ＜ 6 | 213 (26.8%) | 209 (26.7%) | 4 (11.1%) |  |
| ≧ 6 | 583 (73.2%) | 553 (70.8%) | 30 (83.3%) |  |
| CAC score ≧100 | 103 (12.6%) | 90 (11.5%) | 13 (36.1%) | <0.001 |
| CAC score ≧400 | 40 (4.9%) | 32 (4.1%) | 8 (22.2%) | <0.001 |
| CAD-RADS ≧3 | 78 (9.6%) | 70 (8.9%) | 8 (22.2%) | 0.008 |
| Vulnerable plaque (s) | 119 (14.6%) | 108 (13.8%) | 11 (30.5%) | 0.005 |
| Abbreviation: BMI: body mass index; HDL-C: high density lipoprotein-cholesterol; LDL-C: low density lipoprotein-cholesterol; HbA1c: hemoglobin A1c; eGFR: estimated glomerular filtration rate; CAC: coronary artery calcium; NAFLD: Non-alcoholic fatty liver disease; CAD-RADS: The Coronary Artery Disease-Reporting and Data System. *Reference group: normal, mild and moderate NAFLD groups (severe NAFLD vs. reference group). | | | | |
